# Demonstration of a quantum error detection code using a square lattice of four superconducting qubits

**DOI:** 10.1038/ncomms7979

**Published:** 2015-04-29

**Authors:** A.D. Córcoles, Easwar Magesan, Srikanth J. Srinivasan, Andrew W. Cross, M. Steffen, Jay M. Gambetta, Jerry M. Chow

**Affiliations:** 1 IBM T.J. Watson Research Center, Yorktown Heights, New York 10598, USA

## Abstract

The ability to detect and deal with errors when manipulating quantum systems is a fundamental requirement for fault-tolerant quantum computing. Unlike classical bits that are subject to only digital bit-flip errors, quantum bits are susceptible to a much larger spectrum of errors, for which any complete quantum error-correcting code must account. Whilst classical bit-flip detection can be realized via a linear array of qubits, a general fault-tolerant quantum error-correcting code requires extending into a higher-dimensional lattice. Here we present a quantum error detection protocol on a two-by-two planar lattice of superconducting qubits. The protocol detects an arbitrary quantum error on an encoded two-qubit entangled state via quantum non-demolition parity measurements on another pair of error syndrome qubits. This result represents a building block towards larger lattices amenable to fault-tolerant quantum error correction architectures such as the surface code.

## Abstract (web summary)

The physical realization of a quantum computer requires built-in error-correcting codes that compensate the disruption of quantum information arising from noise. Here, the authors demonstrate a quantum error detection scheme for arbitrary single-qubit errors on a four superconducting qubit lattice.

## Graphical abstract

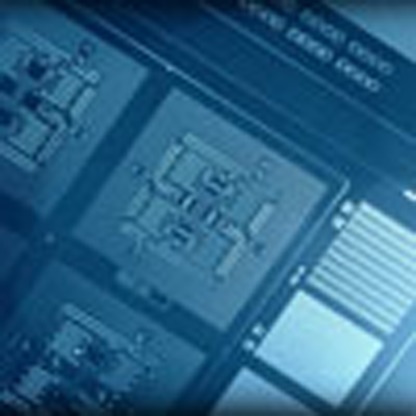

Errors are inevitable in any real information processor. Quantum computers are particularly susceptible to errors as quantum systems are highly sensitive to noise effects that can be exotic compared with the simple bit-flip errors of classical computation. As such, realizing a fault-tolerant quantum computer is a significant challenge that requires encoding the information into a quantum error-correcting code. To add to the difficulty, direct extraction of the information typically destroys the system, and ancillary syndrome systems must be employed to perform non-demolition measurements of the encoded state. Previous work in nuclei^1^,^2^,^3^, trapped ions^4^,^5^,^6^ and superconducting qubits^7^ has attempted to address similar problems; however, these implementations lack the ability to perform fault-tolerant syndrome extraction, which continues to be a challenge for all physical quantum computing systems.

The surface code (SC)^8^,^9^ is a promising candidate to achieve scalable quantum computing due to its nearest-neighbour qubit layout and high fault-tolerant error thresholds^10^. The SC is an example of a stabilizer code^11^, which is a code whose state is uniquely defined by the measurement of a set of observables called stabilizers. Code qubits in the SC are placed at the vertices of a two-dimensional array and each stabilizer involves four neighbouring code qubits. The SC stabilizers are, therefore, geometrically local and can be measured fault tolerantly with a single syndrome qubit^12^. Error detection on a lattice of code qubits is achieved through mapping stabilizer operators onto a complementary lattice of syndrome qubits, followed by classical correlation of measured outcomes. Among the syndrome qubits, a distinction is made between bit-flip syndromes (or *Z*-syndromes) and phase-flip syndromes (or *X*-syndromes). Each code qubit in the SC is coupled with two *X*-syndrome qubits and two *Z*-syndrome qubits, and, in turn, each syndrome qubit is coupled with four code qubits.

Superconducting qubits have become prime candidates for SC implementation^13^,^14^, especially with continuing improvements to coherence times^15^,^16^,^17^ and quantum gates^18^. Furthermore, implementing superconducting resonators as quantum buses to realize the circuit quantum electrodynamics architecture permits a straightforward path for building connectivity into a lattice of superconducting qubits^14^. There are numerous ways of building the SC lattice with superconducting qubits and resonators. Here we employ an arrangement in which each qubit is coupled with two bus resonators and each bus couples with four qubits^14^. Although previously the engineered dissipation of a resonator has been used to stabilize the entanglement of two superconducting qubits to which it is coupled^19^, it is of note that here the stabilization is achieved via explicitly mapping code qubit stabilizers onto syndrome qubits.

Here we experimentally demonstrate the complete algorithm constituting a quantum error detection code that detects arbitrary single-qubit errors in a non-demolition manner via syndrome measurements. The scheme is implemented in a two-by-two lattice of superconducting qubits that represents a primitive tile for the SC. Stabilizer measurements, ubiquitous to fault-tolerant quantum error-correcting codes, are successfully demonstrated in this work for both bit- and phase-flip errors on an encoded codeword. The non-demolition nature of the protocol is verified by demonstrating the preservation of the entangled state constituting the codeword through high-fidelity syndrome measurements in the presence of an arbitrary applied error. These error detection experiments constitute a key milestone for SC implementation, as our operations now extend into the plane of the two-dimensional surface and we show the ability to concurrently perform bit- and phase-parity checks. Moreover, our results illustrate the ability to build structures of superconducting qubits, which are not co-linear but latticed while preserving high-fidelity operations. Moving forward, on improving the measurement and gate fidelities in these systems, further expanding the lattice will lead to important studies of different error-correcting codes and the encoding of logical qubits, thereby allowing experimental investigation of fault-tolerant quantum computing. Our results bolster the prospect of employing superconducting qubit lattices for large-scale fault-tolerant quantum computing.

## Results

### Physical device and quantum control

**Figure 1:**
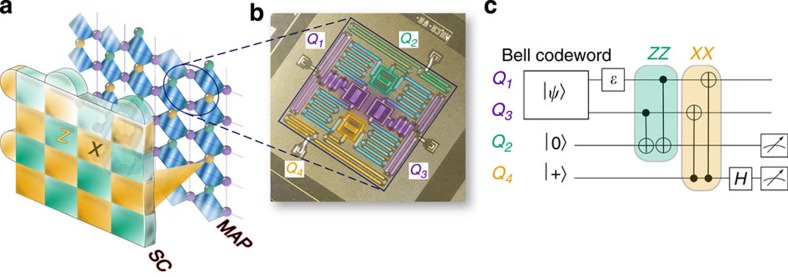
Surface code implementation and error detection quantum circuit. (**a**) Cartoon schematic of SC consisting of alternating square tiles of *X*- (yellow) and *Z*- (green) plaquettes for detecting phase-flip (*Z*) and bit-flip (*X*) errors, respectively. Semi-circular pieces reflect parity checks at the boundaries of the lattice. These plaquette tiles can be mapped onto a lattice of physical superconducting qubits with appropriate nearest-neighbour interconnectivity, as shown in the layer labelled MAP. Here there are code qubits (purple spheres), *X*-syndrome qubits (yellow) for phase parity detection of surrounding code qubits, and *Z*-syndrome qubits (green) for bit parity detection of surrounding code qubits. The physical connectivity for superconducting qubits can be realised via coupling every qubit to two quantum bus resonators, shown as wavy blue diamonds in the MAP. The device studied in this work (false-colored optical micrograph in **b**) embodies two half-plaquettes of the SC as circled in **a**, and allows for independent and simultaneous detection of *X* and *Z* errors on two-code qubits, shaded purple in **b** and labelled *Q*_1_ and *Q*_3_. (**c**) The circuit to implement the half-plaquette operations encodes the bit (*ZZ*) and phase (*XX*) parities of the two-code qubits' Bell state 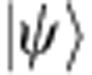 onto the respective syndrome qubits, *Q*_2_ (green) and *Q*_4_ (yellow). Arbitrary errors *ɛ* are intentionally introduced on the code qubit *Q*_1_ and detected from the correlated measurement of the syndrome qubits. *Q*_2_ (*Q*_4_) is initialized to 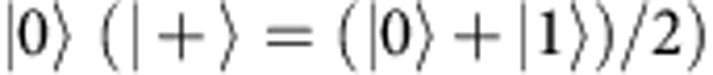. A Hadamard operation, *H*, is applied to *Q*_4_ before measurement.

Our physical device (Fig. 1a,b) consists of a 2 × 2 lattice of superconducting transmons, with each coupled with its two nearest neighbours via two independent superconducting coplanar waveguide (CPW) resonators serving as quantum buses (Fig. 1b; blue). Each qubit is further coupled with an independent CPW resonator for both qubit control and readout. Dispersive readout signals for each qubit are amplified by distinct Josephson parametric amplifiers (JPAs) giving high single-shot readout fidelity^20^,^21^. We implement two-qubit echo cross-resonance (ECR) gates^22^, ECR=*ZX*_90_–*XI*, which are primitives for constructing controlled-NOT (CNOT) operations. Given the latticed structure of our device, we implement four different such gates, ECR^*ij*^ between qubits *Q*_*i*_ (control) and *Q*_*j*_ (target), with *ij*∈{12,23,34,41}. In this arrangement, we use *Q*_1_ and *Q*_3_ (Fig. 1b; purple) as code qubits, *Q*_2_ as the *Z*-syndrome qubit (Fig. 1b; green) and *Q*_4_ as the *X*-syndrome qubit (Fig. 1b; yellow). All ECR gates are benchmarked^22^ with fidelities between 0.93 and 0.97 (for further details see Methods). Single-qubit gates are benchmarked to fidelities above 0.998 with <0.001 reduction in fidelity due to crosstalk, as verified via simultaneous randomized benchmarking^23^. The four independent single-shot readouts yield assignment fidelities (see Methods) all above 0.94. These and other relevant system experimental parameters including qubit frequencies, anharmonicities, energy relaxation and coherence times are further discussed in the Methods.

### Quantum error detection protocol implementation

Our four-qubit square lattice is a non-trivial cut-out of the SC layout (circled in Fig. 1a), and can be used to demonstrate both the *ZZ* and *XX* parity check. The *XX* (*ZZ*) stabilizer is measured by the *X*-syndrome (*Z*-syndrome) qubit. Although previous work^14^,^24^,^25^,^26^ implemented parity checks on linear arrangements of qubits, our experiment goes beyond into the other planar dimension. The extra dimension allows us to demonstrate the [[2,0,2]] code, which contains 2 physical qubits, 0 logical qubits (and hence a single fixed code state), and has a distance of 2, which means arbitrary single-qubit errors are detectable. The codeword is the two-qubit entangled state 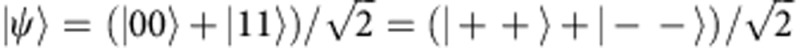, which is protected from any single-qubit error on the codespace via syndrome detection. An arbitrary single-qubit error revealed in the stabilizer syndrome as a bit- (phase) flip simply maps 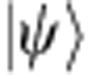 to a negative eigenstate of *ZZ* (*XX*), and a joint bit- and phase-flip (*Y* rotation) maps 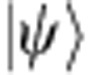 to the negative eigenstate of both *ZZ* and *XX*. By encoding both the *XX* and the *ZZ* stabilizers in the four-qubit lattice, we can protect a maximally entangled state of the two-code qubits against an arbitrary error.

To demonstrate the SC sub-lattice stabilizer measurement protocol (Fig. 1c), we first prepare the two-code qubits in codeword state 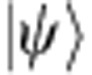, which is a maximally entangled Bell state. Subsequently, the *ZZ* stabilizer is encoded onto the *Z*-syndrome qubit *Q*_2_, which is initialized in the ground state 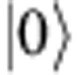. The *XX* stabilizer is encoded onto the *X*-syndrome qubit *Q*_4_, which is initialized in the 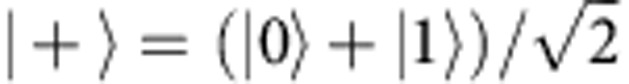 state. Since we perform measurements of the syndrome qubits in the *Z* measurement basis, *Q*_4_ also undergoes a Hadamard transformation *H* right before measurement. The complete circuit as shown in Fig. 1c will detect an arbitrary single-qubit error *ɛ* to the code qubits via the projective measurements of the syndrome qubits. We choose to apply the error on *Q*_1_, but there is no loss of generality if applied on *Q*_3_ instead. Each of the four possible outcomes of the syndrome qubit measurements projects the code qubits onto one of the four maximally entangled Bell states. If no error is present in the sequence, the syndrome qubits are both found to be in their ground state after the measurement, and the prepared codeword state of the code qubits is preserved.

In our experiment, since the two-code qubits (*Q*_1_ and *Q*_3_) are non-nearest neighbours in the lattice, the preparation of the codeword state is performed via two-qubit interactions with a shared neighbouring qubit, *Q*_2_. The gate sequence for this state preparation can be compiled together with portions of the *ZZ* stabilizer encoding. The resulting complete gate decomposition of the circuit from Fig. 1c in terms of our available single- and two-qubit ECR gates is described in detail in the Methods.

**Figure 2:**
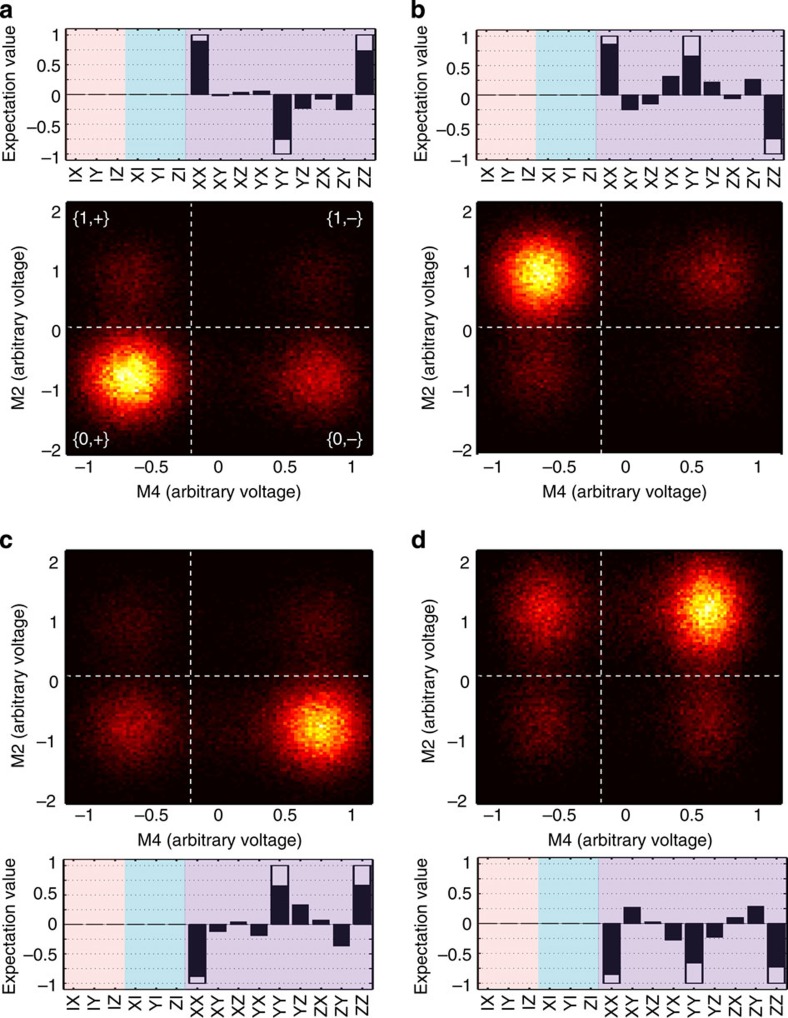
Correlated syndrome single-shot histograms and quantum state tomography of code qubits. The quantum state of the syndrome qubits reveals the entangled state of the code qubits. The colormaps show the single-shot histograms of the syndrome measurements on *Q*_2_ and *Q*_4_. The dashed white lines indicate the threshold used to condition the reconstruction of the code qubit states, represented by a Pauli vector. The pink-, blue- and purple-shaded regions signify *Q*_1_, *Q*_3_ and joint Pauli operators, respectively (black filled bars, experiment, white bars in background, ideal). Each of the possible four outcomes of correlated single-shot measurements of the syndrome qubits is mapped onto one of the four maximally entangled Bell states of the code qubits. Since we always prepare the code qubits in the codeword state 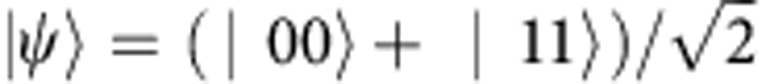 at the beginning of the quantum process, when no error is applied to *Q*_1_, state tomography of *Q*_1_ and *Q*_3_ conditioned on outcomes in the lower left quadrant {0,+} of the colormap recover the same state with fidelity 0.8491±0.0005 (**a**). Introducing an error *ɛ* equal to *X* (**b**), *Z* (**c**) and *Y* (**d**) on *Q*_1_, and conditioning on outcomes in the upper left {1,+}, lower right {0,−} and upper right {1,−} quadrants results in the code qubits reconstructed as 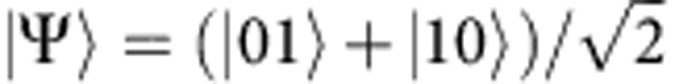 (fidelity 0.8195±0.0006), 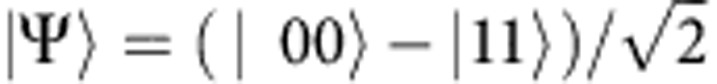 (with fidelity 0.8046±0.0005) and 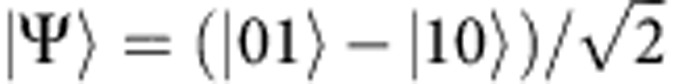 (fidelity 0.8148±0.0006), respectively. The *X*-syndrome qubit, *Q*_4_, is found in its excited state when a phase-flip error has occurred (**c**,**d**), whereas the *Z*-syndrome qubit, *Q*_2_, is found in its excited state as a result of bit-flip errors (**b**,**d**). The quoted uncertainties in reconstructed state fidelities are statistical (see Methods), but we note that systematic errors due to coherence time fluctuations, state preparation and measurement errors can lead to indifelity ∼0.01–0.02.

To implement arbitrary errors to the entangled code qubit state, we apply single-qubit rotations to *Q*_1_ of the form *ɛ*=*U*_*θ*_, where *U* defines the rotation axis and *θ* is the rotation angle (when no angle is given it is assumed *θ*=*π*). Following the error detection protocol of Fig. 1c, we acquire single-shot measurements of the syndrome qubits and correlate independent measurements around various axes of the code qubits for quantum state tomography^27^. First, for the case where *ɛ*=*U*_0_, when no error is added, the two syndrome qubits *Q*_2_ and *Q*_4_ should both be measured to be in their ground states, and from correlating their single-shot measurements, *M*_2_ and *M*_4_, we detect the colour map as shown in Fig. 2a. Here, we can clearly see that a majority of the resulting measurements are located in the lower left quadrant, and we will use the notation {*M*_2_,*M*_4_}={0,+}, with both syndromes signalling a ground state detection (note that measuring *Q*_4_ in the ground state signals a 
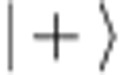
 detection given the *H* before measurement). Conditioned on {0,+}, state tomography of the code qubits is performed, with a reconstructed final state (Pauli vector shown in Fig. 2a), commensurate with the originally prepared codeword state with a fidelity of 0.8491±0.0005. Next, for the case of a bit-flip error to *Q*_1_, or *ɛ*=*X*_*π*_, the resulting syndrome histograms are shown in the colour map in Fig. 2b, where a majority of results are consistent with {1,+}, where the *Z*-syndrome *Q*_2_ is excited to 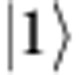 and the *X*-syndrome *Q*_4_ remains in its ground state. Conditioned on {1,+}, the reconstructed final state Pauli vector of the code qubits is now 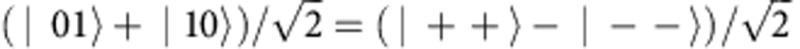, verifying the bit-flip parity error. Then, for the case of a phase-flip error on *Q*_1_, or *ɛ*=*Z*_*π*_, we find that the syndromes give {0,−}, with the *X*-syndrome having changed its state (Fig. 2c). Similarly, conditioned on {0,−}, the code qubit state agrees with 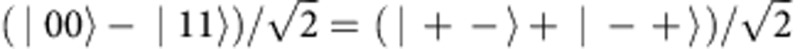, showing the phase flip. Finally, an error *ɛ*=*Y*_*π*_ results in both syndromes flipped, {1,−}, as shown in Fig. 2d with corresponding code qubit Pauli vector in agreement with both a bit and phase flip of the original codeword state 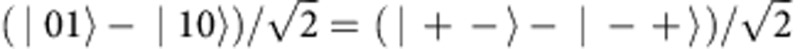.

The reconstructed states reveal important information about our system. First, the measured state fidelity (∼0.80–0.84) is higher than expected (∼0.75) from the measured fidelities of the five two-qubit gates and two independent single-shot measurements. This is because the gates used to prepare the codeword state do not contribute to the accumulated state fidelity loss, but rather reveal themselves as measurement errors. Second, the reconstructed conditional states have little to no weight in the single-qubit subspace. This suggests that in our system there are negligible crosstalk errors (as expected since the code qubits are not directly connected via a bus).

### Tracking arbitrary errors

**Figure 3:**
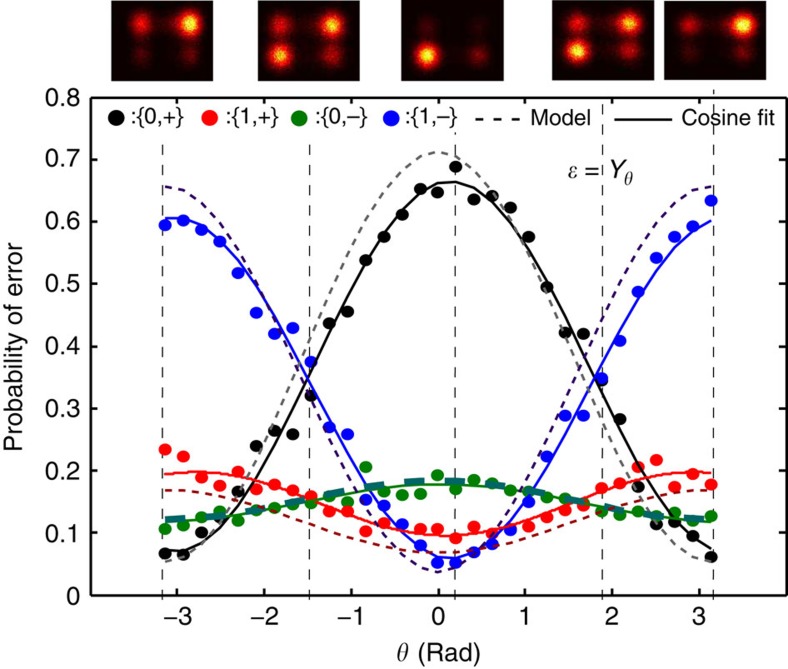
Syndrome qubits single-shot correlated measurement for different *Y*-error magnitudes. The magnitude of each type of error in the code qubits can be extracted from the correlated single-shot traces of the syndrome qubits. Errors of *ɛ*=*Y*_*θ*_, with *θ∈*[−*π*,*π*] are detected by both syndromes, as *Y* errors can be decomposed into a combination of bit- and phase-flip errors. As the magnitude of the *Y* error increases from 0 to *π*, the majority of the outcomes of the syndrome qubits changes from {*M*_2_,*M*_4_}={0,+} (black dots) to {*M*_2_,*M*_4_}={1,−} (blue dots), while the states {*M*_2_,*M*_4_}={1,+} (red dots) and {*M*_2_,*M*_4_}={0,−} (green dots), which indicate pure bit- and phase-flip errors, respectively, remain low probability. Solid lines are simple cosine fits to the data. Dashed lines are master-equation simulations that take into account the measured coherence times and assignment fidelities. Histograms of the correlated single-shot syndrome qubit measurements are shown in the density plots on top for θ∼{−*π*,−*π*/2,0,*π*/2,*π*}, as indicated by the vertical dashed lines, with the syndrome states corresponding to |θ|=*π*/2 showing significant populations in two quadrants.

We can track the outcome of the syndrome qubits as we slowly vary *θ* in an applied error *ɛ*=*Y*_*θ*_ between −*π* and +*π* (see Fig. 3). The state population of the four syndrome qubit states, {0,+} (black dots), {1,+} (red dots), {0,−} (green dots) and {1,−} (blue dots), obtained from the (normalized) number of counts in the correlated histograms conditioned on a readout threshold extracted from calibration measurements (see Methods), are plotted versus *θ*. For an error induced by a unitary operation, the data is explained by cosines (solid lines in Fig. 3). For *θ* near 0, the ground state, {0,+}, is found in both syndrome qubits as expected, whereas for |*θ|*∼*π*, we recover both the syndrome flips {1,−}. The observed contrast between the different syndrome qubit state populations, near 0.6 in Fig. 3, is commensurate with a master-equation simulation that takes into account the measured coherence times of our qubits and the assignment fidelities of the readouts (dashed lines in Fig. 3). Similarly, varying *θ* for *X* and *Z* rotations are shown in the Methods.

**Figure 4:**
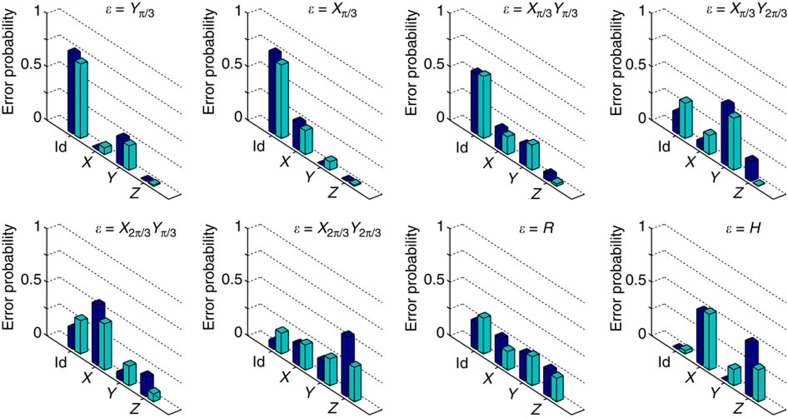
Detection of arbitrary errors. The probability of each type of error, identity (Id), *X*, *Y* or *Z*, is extracted from the correlated syndrome measurements for all the applied *ɛ*, as indicated above each panel. Dark blue bars represent the ideal outcome for each *ɛ* and teal bars are measurements calibrated by the full *X*, *Y* and *Z* error rotation curves. The errors labelled as *R* and *H* correspond to a *Y*_*π*/2_*X*_*π*/2_ operation, which maps the *x*–*y*–*z* axes in the Bloch sphere to *y*–*z*–*x*, and the Hadamard gate, respectively. The results are consistent with a higher uncertainty in the phase-flip error detection, likely due to decoherence during the full sequence and the order of syndrome detection.

To demonstrate arbitrary error detection, we construct *ɛ* via combinations of *X* and *Y* errors. Each panel of Fig. 4 shows a teal bar plot reflecting the experimentally extracted population of each of the four possible syndrome qubit measurement outcomes for the set of errors {*Y*_*π*/3_, *X*_*π*/3_, *X*_*π*/3_*Y*_*π*/3_, *X*_*π*/3_*Y*_2*π*/3_, *X*_2*π*/3_*Y*_*π*/3_, *X*_2*π*/3_*Y*_2*π*/3_, *R*, *H*}, where *R*=*Y*_*π*/2_*X*_*π*/2_ and *H* is the Hadamard operation. Overall, we find decent agreement between the experiment and ideal population outcomes (dark blue bars). The measured populations are renormalized by the observed contrast at *θ*∼0 in Fig. 3, and in the equivalent plots for *X* and *Z* errors shown in the Methods, to account for relaxation and decoherence fidelity loss. Although this renormalization provides an overall fairer comparison with the ideal case, it tends to increase the uncertainty in the bars, especially for the *Z* error due to the loss of contrast and larger data scatter observed for the *X*-syndrome qubit *Q*_4_ (see Methods). This diminished contrast is due to the fact that the *XX* stabiliser is encoded last in our sequence and therefore suffers more from decoherence.

## Discussion

We have provided a set of experiments that demonstrate the detection of arbitrary single-qubit quantum errors on a square lattice of qubits. The experiments combine a variety of key components required for scaling quantum systems up to larger numbers of qubits: high-fidelity one- and two-qubit gates, high single-shot assignment fidelities allowing for non-demolition measurements of code qubits and improved system design to minimize crosstalk effects in non-trivial lattices of nearest-neighbour-coupled qubits. Moving forward, continued improvement of gate and assignment fidelities will be required to reach fault-tolerance thresholds. This will require continued understanding of potential sources of error in our system, such as calibration and crosstalk effects, as well as improved system design and engineering. In addition, achieving shorter measurement times and measurement repeatability will be key for demonstrating large-scale experimental quantum error correction. While there remain significant challenges in implementing complex operations on larger lattices of qubits, the work we have presented here demonstrates that a high degree of control and microwave hygiene can be achieved with superconducting qubits arranged in geometries useful for fault-tolerant quantum computation.

## Methods

### Device fabrication and parameters

The device is fabricated on a 720-μm-thick Si substrate. The superconducting CPW resonators, the qubit capacitors and coupling capacitors are defined in the same step via optical lithography. Reactive ion etching of a sputtered 200-nm-thick Nb film is used to make this layer. The Josephson junctions, patterned via electron beam lithography, are made by double-angle deposition of Al (layer thicknesses of 35 and 85 nm) followed by a liftoff process. The chip is mounted on a printed circuit board and wirebonded for signal delivery and crosstalk mitigation.

The four-qubit transition frequencies are *ω*_*i*_/2*π*={5.303,5.101,5.291,5.415} GHz with *i*∈{1,2,3,4}. The readout resonator frequencies are *ω*_*Ri*_/2*π*{6.494,6.695,6.491,6.693} GHz, while the four bus resonators, unmeasured, are designed to be at *ω*_*Bii*_/2*π*={8,7.5,8,7.5} GHz for *ij*∈{12,23,34,41}. All qubits show around 330 MHz anharmonicity, with energy relaxation times *T*_1(*i*)_={33,36,31,29} μs and coherence times 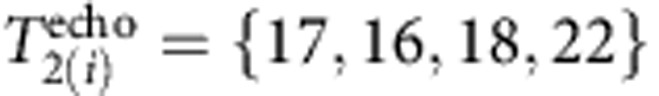 μs. The dispersive shifts and line widths of the readout resonators are measured to be 2*χ*_*i*_/2*π*={−3.0,−2.0,−2.5,−2.8} MHz and *κ*_*i*_/2*π*={615,440,287,1210} kHz, respectively.

### Gate calibration and characterization

Single-qubit gates are 53.3-ns long Gaussian pulses with width *σ*=13.3* *ns. We use single-sideband modulation to avoid mixer leakage at the qubit frequencies in between operations. The sideband frequencies, which are chosen taking into account all qubit frequencies and anharmonicities, are +60, −80, +180 and +100 MHz for *Q*_1_, *Q*_2_, *Q*_3_ and *Q*_4_, respectively. Every single-qubit pulse is accompanied by a scaled Gaussian derivative in the other quadrature to minimize the effect of leakage of information into higher qubit energy levels^28^. All microwave mixers are independently calibrated at the operational frequencies to minimize carrier leakage as well as to ensure orthogonality of the quadratures. Following these calibrations, the single-qubit rotations are tuned by a series of repeated rotations described elsewhere^29^.

Randomized benchmarking (RB) of single-qubit gates^30^ is performed for all four qubits independently and in all possible simultaneous configurations (Table 1). This allows us to establish the degree of addressability error^23^ present in our system. Comparing the individual and simultaneous RB experiments, we can see that the addressability error is 0.001 or lower in all cases.

**Figure 5:**
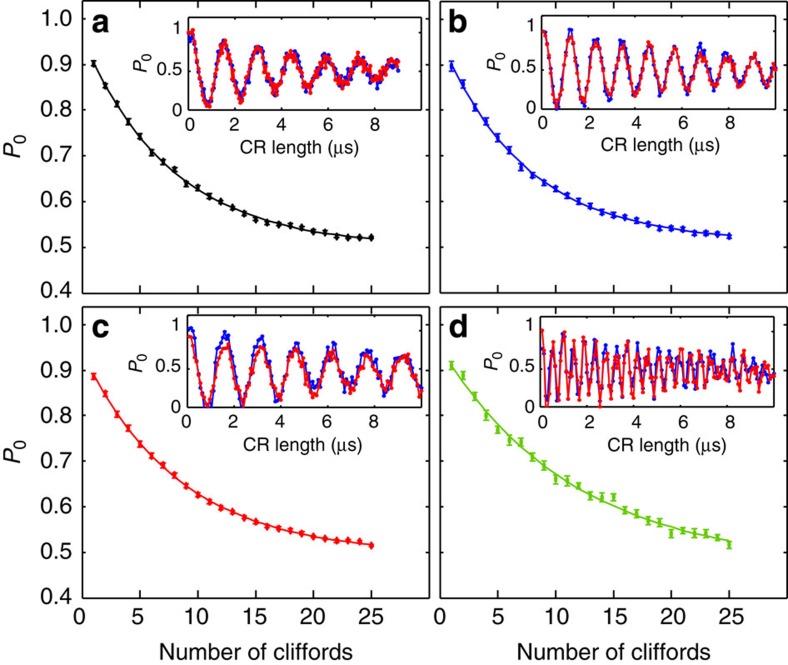
Two-qubit randomized benchmarking. Average population of the ground state of the target qubit, *P*_0_, versus number of two-qubit Cliffords generated via ECR gates between (**a**) *Q*_1_ and *Q*_2_, (**b**) *Q*_2_ and *Q*_3_, (c) *Q*_3_ and *Q*_4_, and (d) *Q*_4_ and *Q*_1_. Each RB experiment is averaged over 50 different sequences. Fits to the experiments are shown as solid lines and yield average errors per two-qubit Clifford of (**a**) 0.0604±0.0006, (**b**) 0.0631±0.0007, (**c**) 0.0569±0.0015 and (**d**) 0.0353±0.0015. Inset shows *ZX* oscillations^22^ of the target qubit state population as a function of the cross-resonance drive length when the control qubit is in the ground (blue) or in the excited (red) state.

The two-qubit ECR gates consist of two cross-resonance pulses of different signs, each of duration *τ*, separated by a *π* rotation in the control qubit. This sequence selectively removes the *IX* part of the Hamiltonian while enhancing the *ZX* term^22^. Each cross-resonance pulse has a Gaussian turn-on and off of width 3*σ* with *σ=*24 ns, included in *τ*. The gates ECR^12^, ECR^23^, ECR^34^ and ECR^41^, where ECR^*ij*^ is the ECR gate between *Q*_*i*_ (control) and *Q*_*j*_ (target), had *τ* of 400, 360, 440 and 190 ns, respectively, for a total gate time of 2 × *τ*+53.3* *ns. We also characterise the two-qubit gates via Clifford RB^22^. Figure 5 shows the RB decays for each of the four gates, yielding an error per two-qubit Clifford gate of 0.0604±0.0006, 0.0631±0.0007, 0.0569±0.0015 and 0.0353±0.0015 for ECR^12^, ECR^23^, ECR^34^ and ECR^41^, respectively.

### Experimental setup

**Figure 6:**
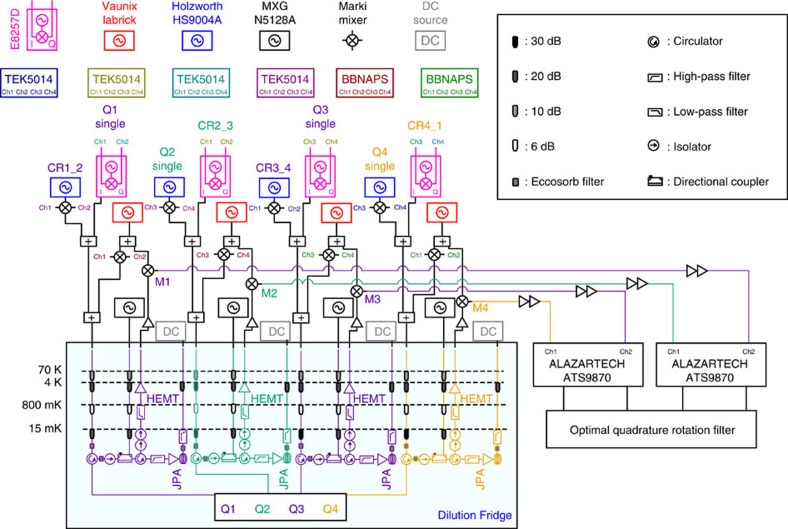
Experimental setup. Detailed wiring scheme for all room temperature control electronics and internal configuration of the Oxford Instruments Triton dilution refrigerator.

**Figure 7:**
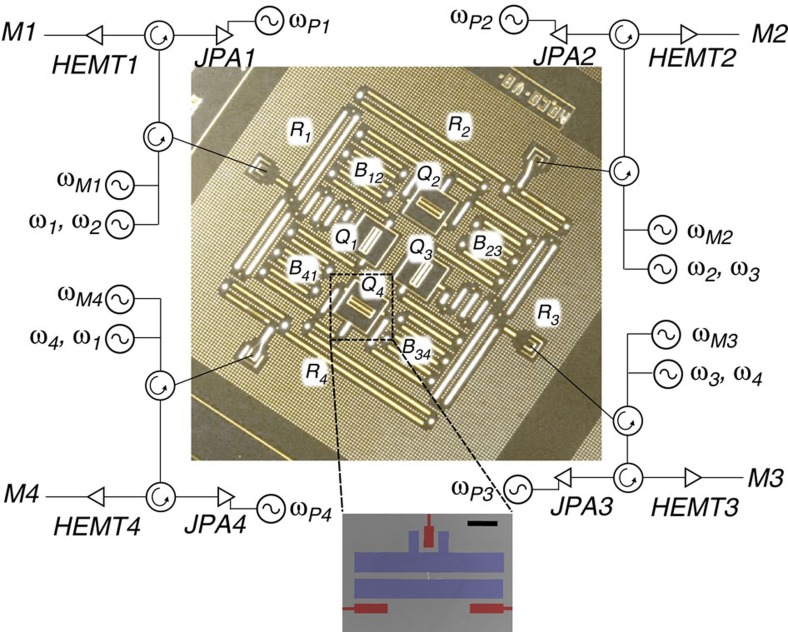
Device and circuit schematic and qubit geometry. The optical image shows all components of the device, including the four qubits, *Q*_1_–*Q*_4_, the four readout resonators *R*_1_–*R*_4_ and the four coupling buses *B*_12_, *B*_23_, *B*_34_ and *B*_41_. The readout resonators also serve as qubit control lines, with single- and two-qubit gates applied at frequencies *ω*_*i*_ with *i*∈{1,2,3,4}. Readout is performed at the resonator frequency *ω*_*Mi*_. Each readout signal is reflected off a JPA, pumped at frequency *ω*_*Pi*_, before being sent to a HEMT amplifier at 4 K. A blowup of one of the qubits is also shown, depicting the capacitor geometry as well as the coupling lines to the readout resonator (green coupler) and to the buses (red couplers). The black scale bar represents a length of 100 μm.

We cool our device to 15 mK in an Oxford Triton dilution refrigerator. Figure 6 shows a full schematic of the measurement setup. We achieve independent single-shot readout for each qubit using a high-electron-mobility transistor (HEMT) amplifier following a JPA (provided by UC Berkeley) in each readout line. The device is protected from environmental radiation by an Amuneal cryoperm shield with an inner coat of Emerson & Cuming CR-124 Eccosorb. All qubit control lines are heavily attenuated at different thermal stages and home-made Eccosorb microwave filters are added at the coldest refrigerator plate. Figure 7 shows the circuit schematic at chip level, including the design of the qubit capacitance and coupling lines.

Single-qubit and two-qubit control pulses as well as resonator readout pulses are generated using single-sideband modulation. The modulating tones are produced by Tektronix arbitrary waveform generators (model AWG5014) for qubit operations. Modulating shapes for readout are produced by Arbitrary Pulse Sequencers from Raytheon BBN Technologies. We either use external Marki I/Q mixers with a Holzworth microwave generator or an Agilent vector signal generator (E8257D) as depicted in Fig. 6. For data acquisition, we use two AlazarTech two-channel digitizers (ATS9870) and the single-shot readout time traces are processed with an optimal quadrature rotation filter^27^.

### Circuit gate decomposition

**Figure 8:**
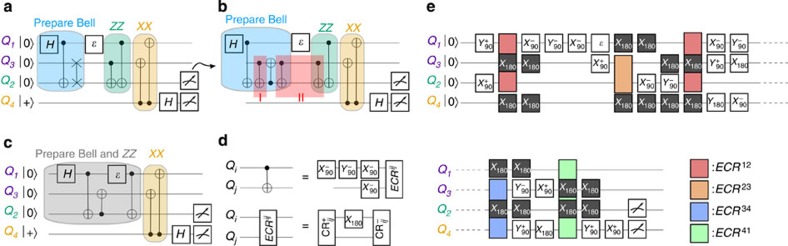
Quantum circuit and CNOT gate decomposition. The *ZZ* and *XX* parity checks are performed on a pair of maximally entangled qubits. This entanglement is achieved in our architecture with one CNOT and one SWAP gate (**a**). The three CNOTs that define the SWAP gate can be combined with the following *ZZ* parity check operation to simplify the circuit and three CNOT gates can be eliminated (**b**). The final circuit implemented in our experiments has a total of five CNOT gates (**c**). We implement our CNOT gates using a simplified version of the 
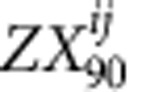
 gate, ECR^*ij*^, consisting of two cross-resonance pulses of different sign separated by a *π* rotation in the control qubit. With that definition, a CNOT gate can be obtained with four single-qubit rotations plus a ECR^*ij*^ operation. An example, not unique, of such decomposition is shown in **d**. The complete gate sequence in our error detection experiments is presented in **e**, where the dark boxes indicate refocus pulses during every two-qubit gate on the two qubits not involved on it.

The circuit in Fig. 1c calls for four two-qubit gates. In addition, the code qubits *Q*_1_ and *Q*_3_ need to be prepared in an entangled state. Since these qubits are not nearest neighbours and there is no provision for interaction between them—a key feature of the SC—we first entangle *Q*_1_ and *Q*_2_ and then perform a swap operation between *Q*_2_ and *Q*_3_ (Fig. 8a). A SWAP gate operation is equivalent to three CNOT gates alternating direction (Fig. 8b). Since two consecutive identical CNOT gates are equal to the identity operation and *Q*_3_ starts from the ground state, the red shadowed regions in Fig. 8 can be omitted. The actual circuit implemented in our experiments is shown in Fig. 8c, where the Bell state preparation and the *ZZ* encoding have been combined.

Our CNOT operations require an entangling gate between the control and the target qubits. We use the ECR^*ij*^ as our CNOT genesis. The ECR^*ij*^ gate plus four single-qubit rotations as depicted in Fig. 8c correspond to a CNOT operation between *Q*_*i*_ and *Q*_*j*_ in our device.

### Tracking bit- and phase-flip errors

**Figure 9:**
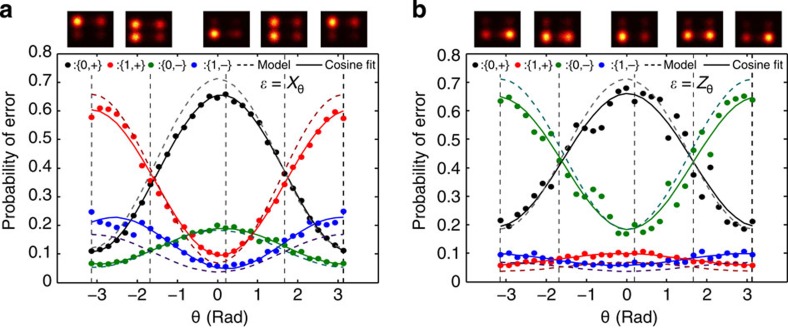
Continuous tracking of pure bit- and phase-flip errors. Errors of *ɛ*=*X*_*θ*_ (**a**) and *ɛ*=*Z*_*θ*_ (**b**) with *θ∈*[−*π*,*π*] are applied to the code qubit *Q*_1_. The syndrome qubit states {*M*_2_,*M*_4_}={0,+} (black), {*M*_2_,*M*_4_}={0,−} (green), {*M*_2_,*M*_4_}={1,+} (red) and {*M*_2_,*M*_4_}={1,−} (blue) indicate the magnitude and nature of the error *ɛ*. Since the *ZZ* and *XX* parities are encoded into *Q*_2_ and *Q*_4_, respectively, pure bit-flip errors are detected by *Q*_2_, whereas pure phase-flip errors are detected by *Q*_4_.

As introduced in the main text (Fig. 3), we can measure the magnitude of the error *ɛ* from the correlated single-shot traces of the syndrome qubits. Here we show the figures complementing Fig. 3 in the main text, corresponding to pure bit-flip error (Fig. 9a) and pure phase-flip error (Fig. 9b). We attribute the increased loss of contrast in the phase-flip error detection (Fig. 9b) to the order of the stabilizer encoding in our circuit, which makes our error detection protocol less sensitive to phase-flip errors.

### Error propagation and syndromes

After the SWAP gate and error in the circuit in Fig. 8a, the state of the code qubits is given by 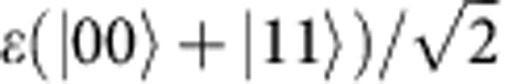, where *ɛ* is some unitary operator acting on the first-code qubit. We will find how the different Pauli errors propagate through the rest of the circuit to produce the different error syndromes.

First, suppose *ɛ* is the bit-flip operation 
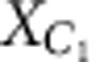
 on the first-code qubit *C*_1_. In this case,


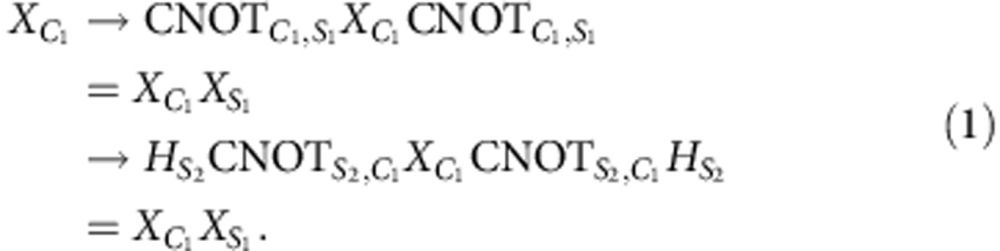


where the sub-indexes *S*_1_ and *S*_2_ refer to the *Z*- and *X*-syndrome qubits, *Q*_2_ and *Q*_4_ in our experiment, respectively. Similarly for the phase-flip operation 
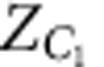
,


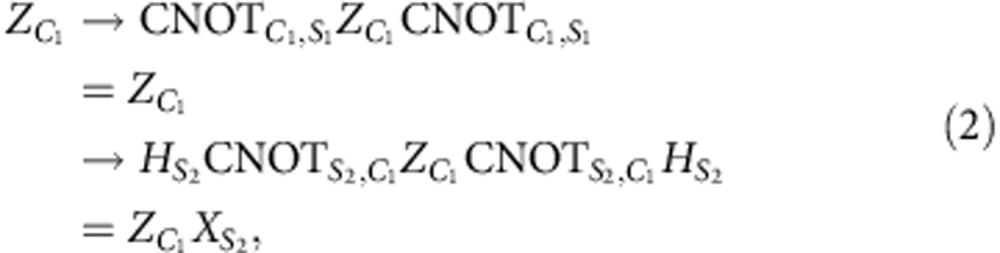


and for 
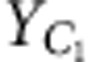



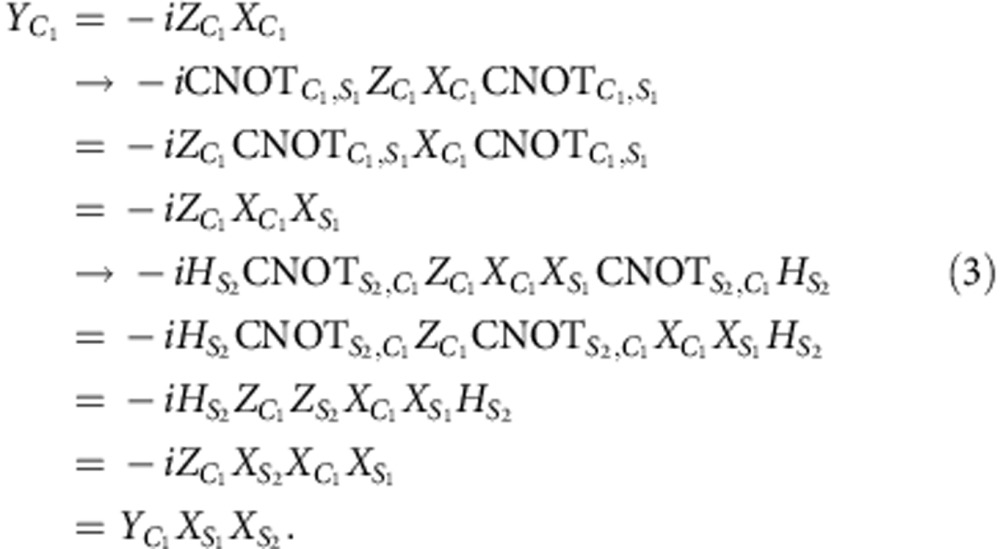


Since the state after the SWAP gate is 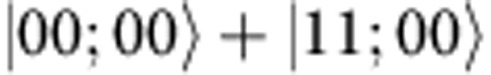 (the qubits are ordered 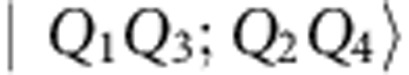 and *Q*_1_, *Q*_3_ are the code qubits), the error syndromes are given by


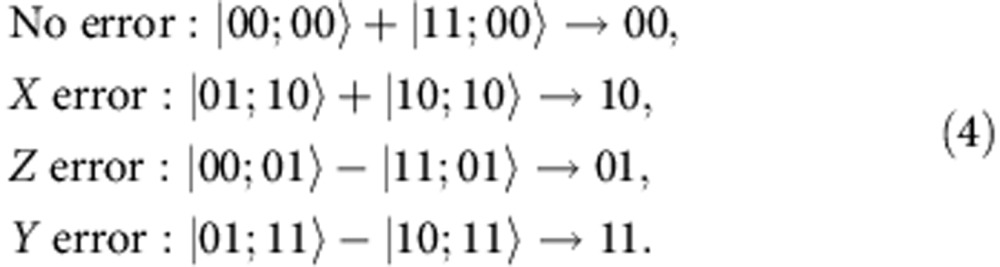


Hence, if the error is a general single-qubit unitary operation


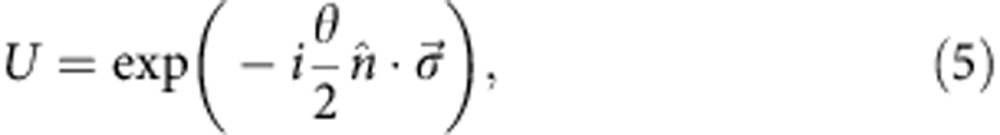


the different error syndromes have the following probabilities of occurring


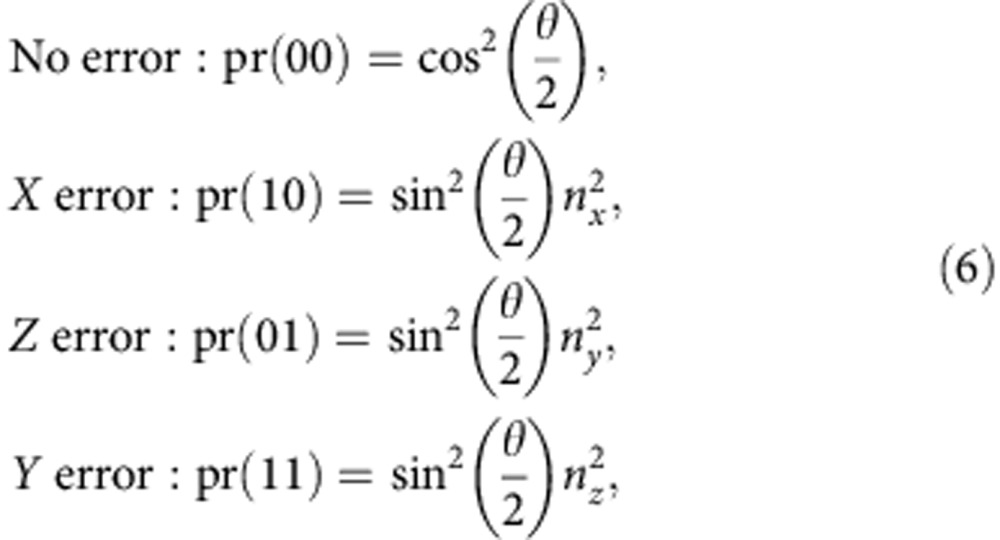


where *n*_*i*_ is the *i*^th^ component of the unit vector.

### Readout characterization

To characterize each readout, we create the 2^4^=16 standard computational basis (calibration) states and record the full time-dependent trajectory of the state of the cavity over a measurement integration time of 3 μs. This process is repeated 19,200 times to gather sufficient statistics. Integrating kernels are obtained for each measurement channel, which extract the full time-dependent readout information^27^. Histograms are fitted to the integrated shots and thresholds for each channel are set at the point of maximum distance between cumulative distributions of the histograms.

The assignment fidelity of each channel is calculated according to the standard formula


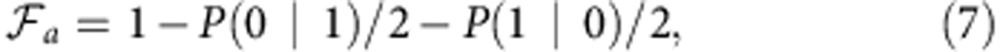


where *P*(0|1) (*P*(1|0)) is the probability of obtaining ‘0' (‘1') when state 
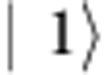
 (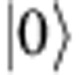) is created. The assignment fidelities are given in Supplementary Table 1.

### State tomography

The conditional states of the code qubits (*Q*_1_ and *Q*_3_) for the different error types (*I*, *X*, *Y* and *Z*) were reconstructed by applying the complete set of 36 unitary rotations 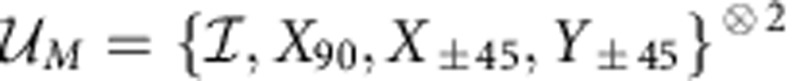 to the state of code qubits to attain a complete set of measurement operators. The fundamental measurement observables 
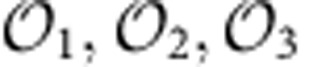
 that are rotated by elements of 
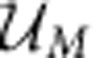
 are constructed from the calibration states by first normalizing the shots for each of the code qubit channels to lie in [−1,1] and then correlating the shots. Note that if the calibrations were perfect, then 
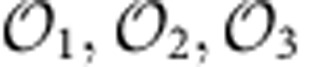
 are equal to *ZI*, *IZ* and *ZZ*.

For each of the 36 different measurement settings, we bin each shot according to the measurement results of the syndrome qubits *Q*_2_ and *Q*_4_. As there are two syndrome qubits, there are four bins labelled by down–down, down–up, up–down and up–up. Denoting the conditional states *ρ*^dd^, *ρ*^du^, *ρ*^ud^ and *ρ*^uu^ and we have full tomographic information of the state of the code qubits for each of the four bins. The shots are correlated to create the expectation values of each conditional state. Hence, for each 
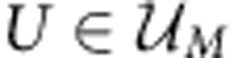
, label *ab*, and observable 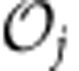, we have an estimate of trace
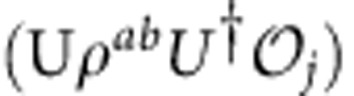
.

For each label *ab*, we have a measurement vector *m*^*ab*^ of length 108 (36 unitary rotations × 3 fundamental observables). Choosing any representation *x*^*ab*^ of *ρ*^*ab*^ in some operator basis allows us to write


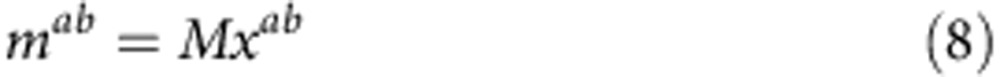


where *M* is a constant matrix whose entries depend only on the choice of operator basis. We choose to use the standard Pauli basis to represent *ρ*^*ab*^,


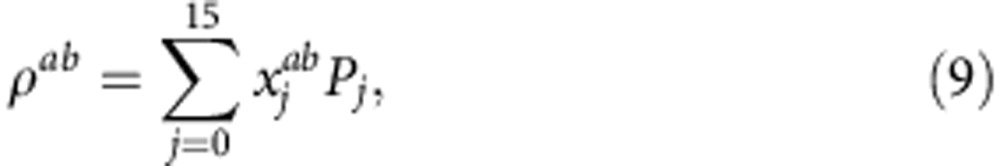


which implies *M* is a 108 × 16 matrix. Enforcing *ρ*^*ab*^ to be trace 1 sets *x*_0_=1/4.

*x*^*ab*^ can be solved for in a variety of ways, the most straightforward of which is linear inversion via computing the pseudoinverse of *M*. While linear inversion provides a valid statistical estimator, it does not enforce positivity of the state. Alternatively, one can maximize the likelihood function for the measurement results under the assumption of Gaussian noise^29^ and solve the following constrained quadratic optimization problem


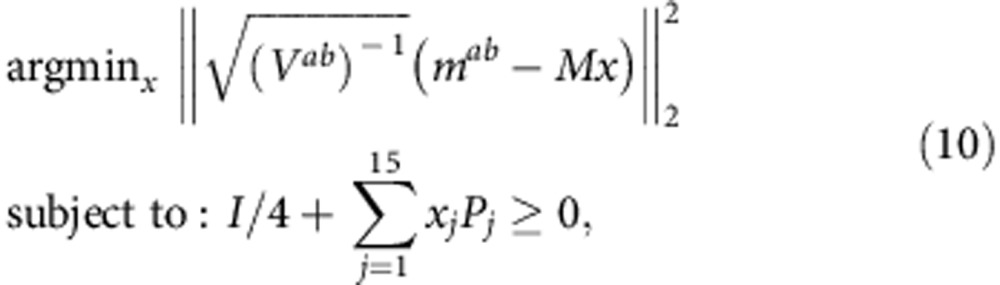


to obtain a physically valid state. Here *V*^*ab*^ is the variance matrix of the measurement matrix. When only Gaussian noise is present, solving this optimization problem is equivalent to finding the closest physical state to the linear inversion estimate^31^.

We quantify the state reconstruction via the state fidelity between *ρ*_noisy_=*ρ*^*ab*^ and the ideal target state 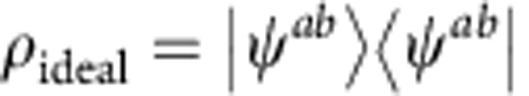;


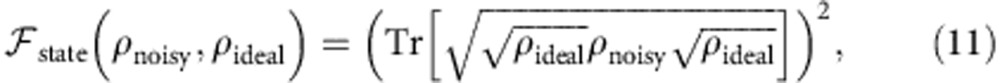


where, as mentioned in the main text, the ideal states for the different syndrome results are given by


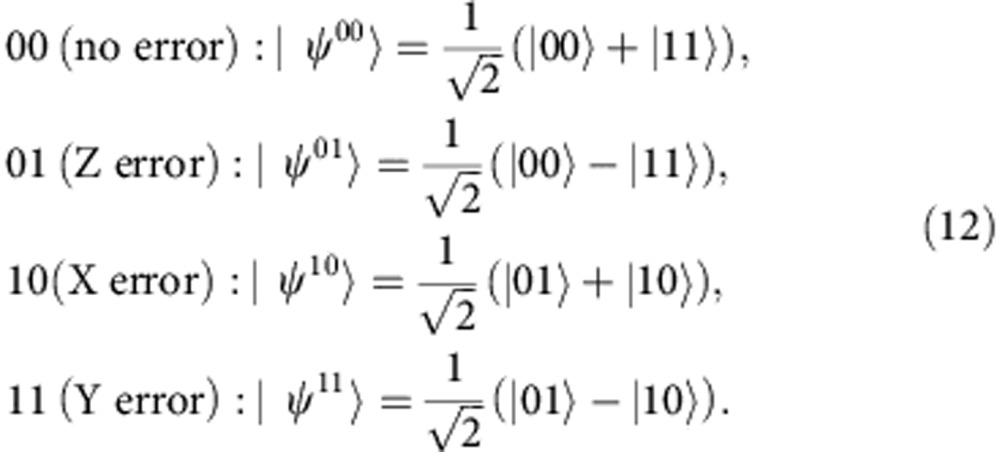


The results are contained in Supplementary Table 2. The variance in the state fidelity is computed via a bootstrapping protocol described in ref. 29 and the physicality is the sum of the negative eigenvalues of the linear inversion estimate. We see that linear inversion produces physical estimates in all cases and there is negligible difference between the fidelities of the physical and linear inversion estimates.

### Insensitivity to state-preparation errors

Since we are conditioning on the measurement results of the syndrome qubits, the error detection circuit has the useful feature that rotation errors on the prepared (encoded) two-qubit state correspond only to decreasing the success probability of preserving the desired state. From a tomographic standpoint, we can accurately reconstruct the conditioned state as long as the total number of shots is large relative to the error syndrome probability, so that sufficient measurement statistics are available.

To make this precise, suppose that the ideal initial state 
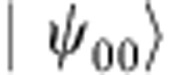
 is rotated via some error operator *E* to the state


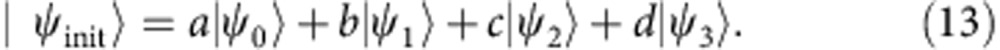


This gives the following syndrome probabilities:


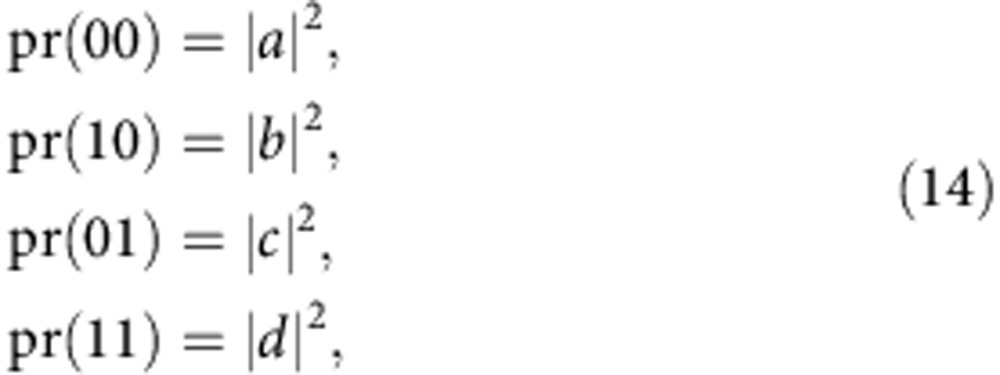


and so the probability of successfully obtaining the correct state is |*a*|^2^. Since the shots producing the error syndromes are evenly distributed throughout the different unitary rotation pulses on the code qubits, the effect on the code state is to reduce the number of shots by a factor of |*a*|^2^, which can also be thought of as a rescaling of the measurement variances by 
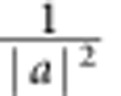
. Hence, for each of the 108 different measurement observables 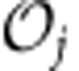, 
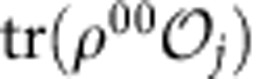
 has a single-shot variance that scales as 
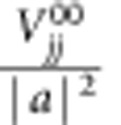
. This implies we expect that, to first order in *θ*, state tomography is robust to over-under rotation errors.

We can model and verify this effect by directly applying a unitary error of varying strength on the first-code qubit. The general unitary Kraus operator is 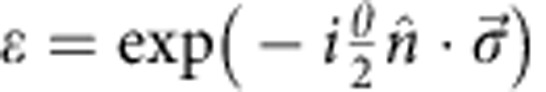 and the probabilities for the different syndromes are given by equation (6). For simplicity, we chose a purely *X* rotation so *ɛ*=cos(*θ*)*I*−*i* sin(*θ*)*X* and varied the size of the angle in 30 steps from −*π* to *π*. The state fidelity 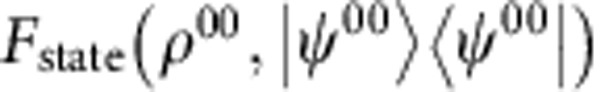 as a function of *θ* is shown in Supplementary Fig. 1. As expected, the first derivative appears to smoothly converge to 0 as *θ* converges to 0 and the loss in fidelity is a result of insufficient statistics for the 00-syndrome state.

This discussion also allows us to more accurately predict the output conditional state fidelities. As demonstrated, we can effectively ignore coherent errors in the first two CNOT gates, since they are used for state preparation and errors in these operations show up as a reduction in the number of shots available for tomography. Assuming the number of shots is large enough, and ignoring single-qubit errors, we are only concerned with errors in the final three CNOT gates. From two-qubit RB, the average gate fidelity of our CNOT gates is ∼0.94. Hence, assuming depolarizing errors, we can obtain an approximate gate fidelity for the comprised circuit of ∼0.94^3^=0.83 and state fidelities with similar values, which is consistent with our obtained fidelities in Supplementary Table 2.

## Author contributions

J.M.C. and J.M.G. designed the experiments. A.D.C. and S.J.S. characterized devices and ran the experiments. A.W.C, J.M.G. and M.S. developed the gate breakdown for the code implementation. E.M., A.D.C. and J.M.G interpreted and analysed the experimental data. All authors contributed to the composition of the manuscript.

## Additional information

**How to cite this article:** Córcoles, A.D. *et al.* Demonstration of a quantum error detection code using a square lattice of four superconducting qubits. *Nat. Commun.* 6:6979 doi: 10.1038/ncomms7979 (2015).

## Supplementary Material

Supplementary Information Supplementary Figure 1 and Supplementary Tables 1-2

## Figures and Tables

**Table 1:** Summary of simultaneous single-qubit RB.

**Qubit label**	***M***_**1**_ **(× 10**^**−3**^)	***M***_**2**_ **(× 10**^**−3**^)	***M***_**3**_ **(× 10**^**−3**^)	***M***_**4**_ **(× 10**^**−3**^)
0001	—	—	—	1.07±0.03
0010	—	—	1.18±0.03	—
0100	—	1.22±0.03	—	—
1000	1.37±0.03	—	—	—
0011	—	—	1.47±0.03	1.15±0.03
0101	—	1.33±0.04	—	1.16±0.02
1001	1.35±0.03	—	—	1.10±0.03
0110	—	1.40±0.04	1.58±0.03	—
1010	1.73±0.05	—	2.06±0.05	—
1100	1.30±0.05	1.35±0.03	—	—
0111	—	1.49±0.03	1.45±0.02	1.18±0.03
1011	1.90±0.07	—	2.22±0.08	1.12±0.03
1101	1.36±0.03	1.41±0.04	—	1.06±0.04
1110	1.90±0.05	1.32±0.03	1.98±0.06	—
1111	2.07±0.05	1.37±0.05	2.12±0.06	1.17±0.04

RB, randomized benchmarking.

The ones in the first column indicate that qubits in the string *Q*_1_*Q*_2_*Q*_3_*Q*_4_ are being randomized and the zeros indicate that the qubits are being left unperturbed.
