# Supplementary material for: Demonstration of a quantum error detection code using a square lattice of four superconducting qubits
Source: Nat Commun. 2015 Apr 29;6:6979. doi: 10.1038/ncomms7979 (PMC4421819; doi:10.1038/ncomms7979)
Supplement: Supplementary Information — Supplementary Figure 1 and Supplementary Tables 1-2 [file ncomms7979-s1.pdf]

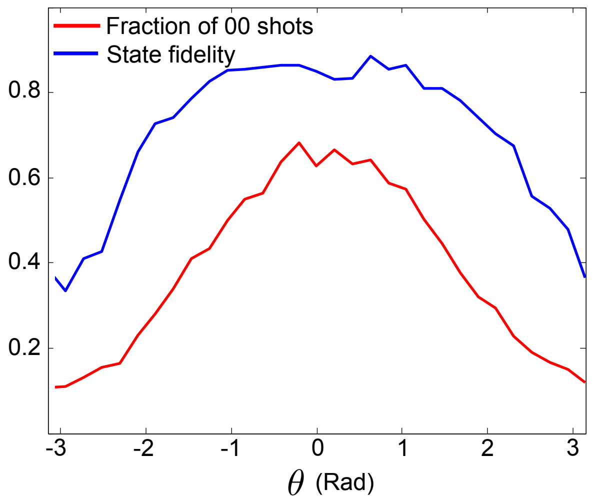

Supplementary Figure 1. **Fidelity and Error Angle. a**, Fidelity and fraction of down-down shots as a function of bit error angle.

Supplementary Table 1. Assignment fidelities of the readout channels

M_1	M_2	M_3	M_4
0.9592	0.9476	0.9416	0.9646

Supplementary Table 2. State Reconstruction Parameters for Different Error Types

Error	Raw Fidelity	Fidelity	Var(Fidelity)	Physicality
None	0.8490	0.8491	0.0018	0
X	0.8194	0.8195	0.0021	0
Y	0.8152	0.8148	0.0022	0
Z	0.8047	0.8046	0.0019	0
